# Surface-Integrated Electric Field Sensor for the Detection of High-Voltage Power Lines

**DOI:** 10.3390/s21248327

**Published:** 2021-12-13

**Authors:** Gunbok Lee, Jeong-Yeon Kim, Gildong Kim, Jae Hee Kim

**Affiliations:** 1 Propulsion System Research Department, Korea Railroad Research Institute (KRRI), Uiwang 16105, Korea; gunbok@krri.re.kr (G.L.); gdkim@krri.re.kr (G.K.); 2 School of Electrical, Electronics and Communication Engineering, Korea University of Technology and Education, Cheonan 31253, Korea; jeongyeonday@koreatech.ac.kr

**Keywords:** electric field sensor, drone, overhead line

## Abstract

When a drone is used for inspection of facilities, there are often cases in which high-voltage power lines interfere, resulting in the drone being caught or falling. To prevent this type of incident, drones must be capable of detecting high-voltage power lines. Typically, a strong electric field is formed around the high-voltage lines. To detect the electric fields around high-voltage lines, this study proposes an electric field sensor that may be integrated within the body of a drone. In a laboratory environment, a voltage of 25 kV was applied to an overhead line, and the induced voltage in the proposed sensor was measured at various electric field intensities. Over an electric field range of 0.5 to 10.1 kV/m, a voltage of 0 to 0.77 V was measured with each proposed sensor. In addition, the electric field and the voltage induced in the sensor were measured in a real-world railway environment with overhead lines. Under these conditions, the proposed sensor has the compensated value of 4.5 when the measured electric field was 4.05 kV/m. Therefore, the proposed sensor may be applied in drones to measure large electric fields and to detect the presence of high-voltage lines in its vicinity.

## 1. Introduction

Recently, more and more drones are used for the inspection of electrical facilities. However, when these drones approach a facility to obtain high-resolution photos, the distances between the power lines and drone are not accurately identified, which may lead to the drone becoming caught in the lines during maneuvers. If the drone becomes caught in a high-voltage power line, the drone may be destroyed; furthermore, the high-voltage lines may be damaged, thereby affecting numerous electrical facilities connected to these high-voltage lines. Therefore, a method for detecting power lines is required when drones are used in the vicinity of high-voltage power lines.

A commonly used method for the detection of high-voltage lines utilizes images or videos taken during the drone operation. This method acquires image data near the drone and extracts information involving the voltage lines using algorithms such as the Hough transform, Radon transform, and Line Segment Detector [1,2]. By extracting this power line information using this method, the presence of power lines near the drone can be detected. However, when detecting these lines using the captured images, it is difficult to determine the exact locations and distances of the lines.

Several previous studies have proposed the measurement of the voltage of high-voltage lines via non-contact methods [3,4,5,6]. However, these methods do not provide information on the location of these high-voltage lines. To obtain voltage measurements for high-voltage lines, the electric field was measured at a specific location and the measured electric field values were converted into voltages. For measurement of the electric field, a D-dot E-field sensor with a parallel distributed electrode was developed, and sensor arrays were constructed to measure the voltage. 

In the case of high-speed railways, power at 60 Hz and 25 kV is supplied to the overhead line. When such a high voltage is applied to the overhead line, a strong electric field is generated around the overhead line. In this study, we propose a method for measuring electric fields such that drones may be able to detect high-voltage lines in their vicinity. Upon measuring an electric field, the presence of high-voltage lines in the surrounding area may be detected using the relative intensity of the measured electric field. Several methods have been proposed to obtain electric field measurement [7,8,9,10,11,12]. The most common method involves the use of a dipole antenna [8]. When a dipole antenna is employed, the varying electric field is detected by the two wires of the dipole antenna. The intensity of the electric field can be estimated by determining the voltage applied across the two wires. However, dipole antenna may only be reduced to a certain size because the magnitude of the detected signals is reduced as the size of the antenna is reduced. If the antenna is small, considerable amounts of noise are included, leading to increased errors in the measured electric field. It has been reported that, for a small dipole antenna, the signal-to-noise ratio (SNR) of the electric field was decreased by four times the scaling factor when the SNR was measured with different antenna lengths [10]. Therefore, it is advantageous to increase the size of the antenna when designing an antenna for measuring electric fields. Recently, a field mill sensor has been proposed to precisely measure the electric field in a high-voltage direct-current transmission line [12]. The measurement results in an actual high-voltage environment using the sensor were presented, and the results were also quite accurate. However, since the size of the proposed sensor is large, it is not a suitable application for drones.

In this study, we proposed a method involving the use of a component of a drone to measure the electric field generated by high-voltage power lines. If the body of the drone is used as an electric field sensor, the size of the sensor can be made large; thus, it has the advantage of increasing the induced voltage of the sensor. Moreover, there is no need to reserve additional space for the electric field sensor. If the electric field is constantly monitored, the presence of high-voltage lines in the vicinity of the drone can be inferred when the measured electric field is higher than a set threshold value. The drone may then avoid high-voltage lines based on the measured electric field values. The remainder of this paper is structured as follows. Section 2 examines the characteristics of general sensors and proposes a structure for the desired sensor such that it can be applied to drones. Section 3 verifies the operation of the sensor under strong electric fields using laboratory and field experiments. Finally, Section 4 outlines the findings and presents the conclusions of this study.

## 2. Electric Field Sensor

### 2.1. Principles of Electric Field Measurements

**Figure 1:**
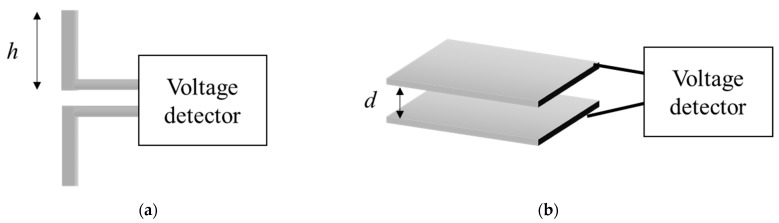
Conventional electric field sensors: (**a**) small dipole; (**b**) parallel plate capacitor.

Electric fields and induced voltages are closely related. The structures most commonly applied for sensors used to measure electric fields are small dipole antenna. In addition, two parallel plates separated by a set distance can also be used as sensors for electric field measurements, although this type of the sensor is not commonly used. Figure 1 shows the structure of a small dipole antenna and that of two parallel plates. In general, when a small dipole is placed where an electric field is generated, the voltage induced in the small dipole can be expressed by the following equation [9,13]:

(1) $$V_{OC}(\omega )=hE(\omega )$$

where *V_OC_* is the voltage, *E*(*ω*) is the electric field, *ω* is the angular frequency, and *h* is half of the total length of the small dipole antenna.

Similarly, in the case of an electric field sensor composed of two parallel plates, the electric field formed between the parallel plates is directly related to the voltage applied between the two parallel plates. If the distance between the parallel plates is d and the magnitude of the electric field formed between the two parallel plates is *E*(*ω*), then the following relationship holds for the voltage *V_OC_* [14]:

(2) $$V_{OC}\left(\omega \right)=-E\left(\omega \right)d$$

Assuming that the electric field is constant from Equations (1) and (2), it can be seen that the induced voltage increases when the dipole antenna is lengthened or the distance between two parallel plates is increased.

### 2.2. Induced Voltage with Respect to the Direction of the Electric Field Sensor

**Figure 2:**
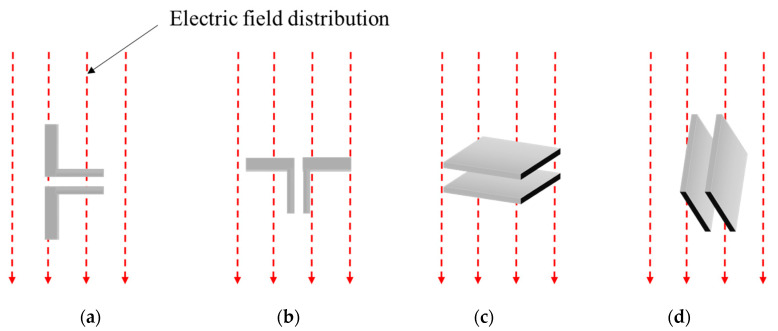
Orientation for the measurement of voltage induced by an electric field: (**a**) dipole (vertical); (**b**) dipole (horizontal); (**c**) parallel plate capacitor (horizontal); (**d**) parallel plate capacitor (vertical).

**Figure 3:**
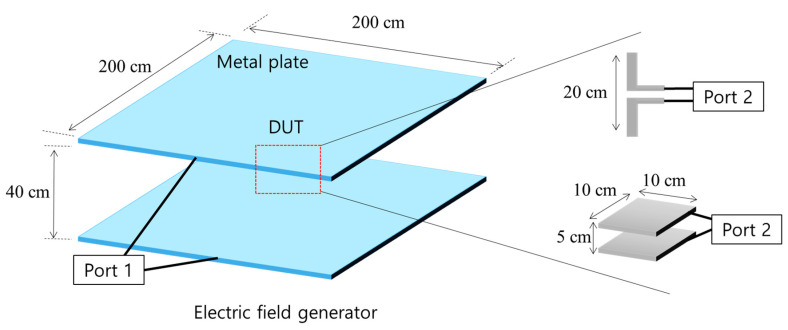
Schematic of the structure used for the simulation of the induced voltage with respect to the orientation of the sensor.

When a dipole and a parallel plate capacitor are used at a location with a uniform distribution of an electric field, the magnitude of the induced voltage varies depending on the orientation of the sensors. For the following simulations, the orientation of the sensors was varied, as shown in Figure 2, in a uniform electric field. To simulate the abovementioned configurations, a structure that creates a uniform electric field is required. Therefore, an electric field generator was constructed to generate an electric field of a constant magnitude in a uniform direction. The structure of the electric field generator used two large metal plates, and a voltage was applied. When a voltage is applied to the two plates, a uniform electric field is formed between the plates. Figure 3 shows the structure of the electric field generator and the orientation of the sensor in the simulation. 

**Figure 4:**
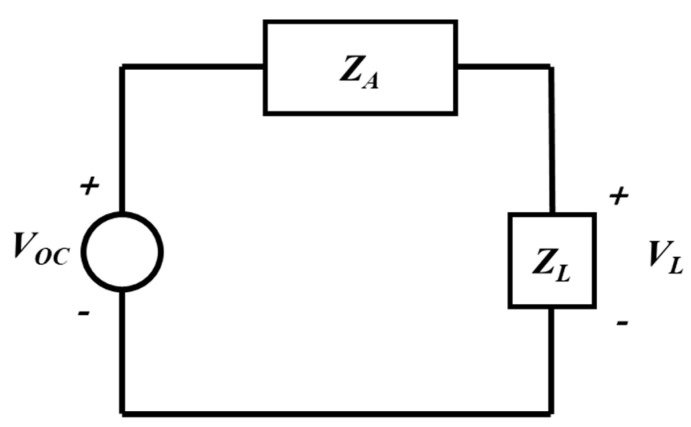
Equivalent circuit model of the conventional electric field sensors.

The horizontal and vertical lengths of the plate were 200 cm, and the distance between the two plates was set to 40 cm. The voltage was applied through port 1, and 1000 V were applied in the simulation. Although the frequency of a real-world overhead power line voltage is 60 Hz, it is difficult to observe clear characteristics under these conditions via simulation because the induced voltage in the sensor is low at frequency of 60 Hz. Because the induced voltage in the sensor is generally proportional to the frequency, a frequency of 60 kHz (1000 times greater than the conventional power line frequency) was used in the simulations. The increase in induced voltage according to the increase in frequency can be derived from the equivalent circuit model. For the structures of Figure 1, the equivalent circuit model can be represented as Figure 4. [13]. The induced voltage of the load impedance *Z_L_*(*ω*) is expressed by the following equation.

(3) $$V_{L}\left(\omega \right)=V_{OC}\left(\omega \right)\frac{Z_{L}\left(\omega \right)}{Z_{A}\left(\omega \right)+Z_{L}\left(\omega \right)}$$

where $Z_{A}\left(\omega \right)$ is the input impedance of the sensors. For the small dipole in Figure 1a, the sensor impedance $Z_{A}\left(\omega \right)$ can be expressed as follows.

(4) $$Z_{A}\left(\omega \right)=-j\frac{\eta_{0}}{2\pi \beta h}\left(\Omega -2-ln4\right)$$

where $\eta_{0}$ is free space wave impedance (120π ohms) and Ω is thickness factor. Ω=2ln(2h/a). where a is the antenna radius and h is the half length of the dipole antenna. $\beta =\omega \sqrt{\mu \varepsilon }$ is the propagation constant. From the Equation (4), If the frequency is high, $Z_{A}\left(\omega \right)$ becomes small. Therefore, the voltage induced by the sensor increases with higher frequency. For the parallel plate capacitor in Figure 1b, the sensor impedance can be simply regarded as the impedance of capacitor. If we assume the capacitance of the structure is *C_P_*, the sensor impedance expressed by the following equation.

(5) $$Z_{A}\left(\omega \right)=-j\frac{1}{\omega C_{P}}$$

From the Equation (5), as the frequency increases, the $Z_{A}\left(\omega \right)$ decreases; thus, the voltage induced in the sensor increases. 

Considering the device under test (DUT), a small dipole antenna and parallel palate capacitor were used, and these were placed in the middle of the electric field generator. The length of the small dipole was set to 20 cm, and the dimensions of the parallel plate capacitor were 20 cm in width and length and 5 cm in height. Next, each sensor was connected to port 2 with a load resistance of 1 MΩ to simulate the induced voltage. The simulations were performed using CST Studio Suite, which is a commercial software package for electromagnetic (EM) analysis. The results of the simulations are presented in Table 1.

By examining the voltages of the sensors induced in the simulations, it can be seen that, in the small dipole antenna, a voltage of 127 V is induced in the vertical orientation, while for the parallel plate capacitor, a voltage of 108 V is induced in the horizontal orientation. No voltage was induced in the horizontally placed dipole antenna or the vertically placed parallel plate capacitor. Therefore, it can be observed that the small dipole antenna and parallel plate capacitor have structures that detect electric fields that are generated in specific orientations.

### 2.3. The Proposed Electric Field Sensor 

**Figure 5:**
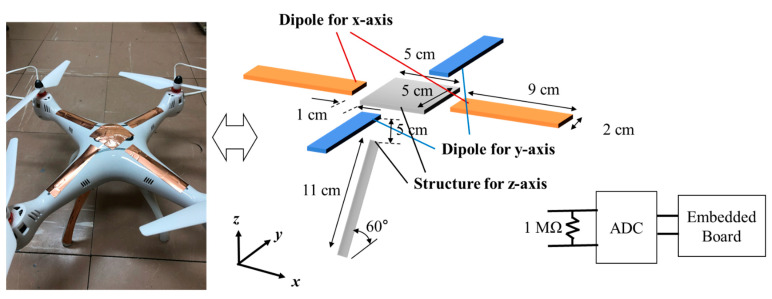
Photo and schematic of the structure of the proposed electric field sensor.

The structure of the sensor for measuring the relative intensity of an electric field is illustrated in Figure 5. Because drone-like structures are not long in the vertical direction, dipole antenna cannot be applied to z-axis. Moreover, it is difficult to make a sufficient induced voltage using only parallel plate capacitor because the height is low. To utilize the body of the drone as much as possible for the sensor, a copper plate of 5 cm in width and length was applied to the upper surface of the drone. A metal post was attached to one side of the drone’s leg. The length of the metal attached to the leg of the drone was 11 cm. The use of the leg of the drone as a part of the sensor structure is advantageous for receiving z-axis electric fields. A dipole antenna structure was used to measure the electric field in the x-axis and y-axis directions. Metal bars with a length of 9 cm and a width of 2 cm were attached to the four frames of the drone. A resistor of 1 MΩ was used to load impedance. This resistance serves to prevent sudden surges in the voltage measurements due to noise generated by the substrate and air. The relative intensity of the electric field can be determined by measuring the voltage across each sensor structures. The analog voltage is converted into a digital voltage using an analog digital converter (ADC), and the final value of the digitally converted signal determined via digital signal processing in the embedded board. A 16 bit ADS1115 was used as the ADC, and analog signal inputs from 0 to 4.09 V were converted into 16 bit digital values. Since a 60 Hz voltage source is used in the power line, the frequency of the electric field was also 60 Hz. Therefore, the data sampling frequency must be higher than 120 Hz (two times 60 Hz) according to the Nyquist sampling theory. The sampling rate of this system was 475 s^−1^. A Raspberry Pi 4.0 board was used as an embedded board for ease of data processing.

The induced voltages for the different orientations of the proposed sensor were simulated. The simulation environment was the same as in Section 2.2. The simulated result is shown in Table 2. ‘Dipole for x-axis’ is a device for measuring the voltage induced from two metals arranged in the x direction. ‘Dipole for y-axis’ refers to the structure of a dipole antenna arranged parallel to the y-axis. ‘Structure for z-axis’ is a structure for measuring the voltage induced between the upper metal plate and the drone’s legs. From the simulation results, the proposed sensor well receives the electric field formed in each direction. However, in the case of the ‘structure for z-axis’, the drone’s leg is curved in the y-axis direction, such that it receives electric fields in both the y-axis and z-axis directions.

In the case of the dipole antenna, it is obvious that the induced voltage decreases as the length becomes shorter. Since the structure for z-axis uses upper metal plates and metal posts, it is necessary to investigate how the induced voltage changes according to the length change in this structure. The induced voltage of the sensor according to the length change is shown in Table 3. For the metal post, which is integrated with the drone’s leg, the induced voltage decreases at a constant rate as the length of the metal post decreases with respect to the electric field on the z-axis and y-axis, respectively. In the case of the upper metal plate, the induced voltage increases as the size increases. In addition, more voltage is induced by the z-axis electric field than by the y-axis electric field. However, since the body size of the drone is fixed, there is a limit to increasing the size of the upper metal plate, and it is necessary to appropriately select the size of the plate in consideration of the size of the drone.

## 3. Results

**Figure 6:**
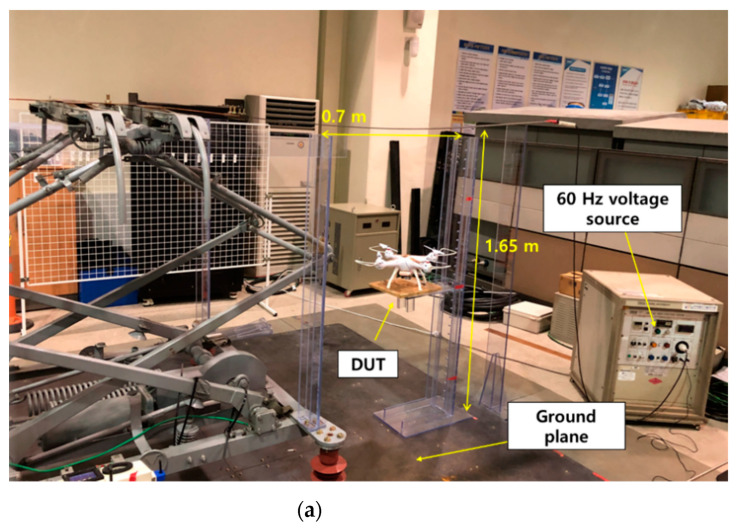

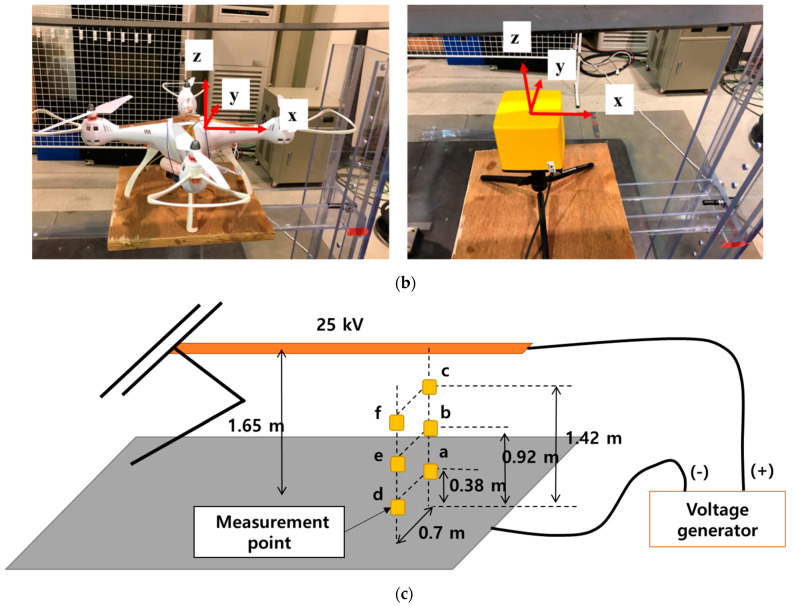
(**a**) Schematic of the experimental setup for electric field generation; (**b**) measurement direction of the sensor; and (**c**) position of the measurement positions.

To measure the voltage induced in the sensor with respect to the electric field, an experimental setup was constructed, as shown in Figure 6. This setup reproduced a railway environment with overhead lines. Figure 6a shows the overall configuration of the experimental setup, which consisted of a ground plane, overhead line, and voltage source. Typically, a high voltage of 60 Hz and 25 kV is supplied in the overhead lines of railway environments. Therefore, a voltage source of 60 Hz was used. In the experiment, the generated voltage was in the range of 1 to 25 kV. For the ground plane, a wide metal plate was used. The wide metal plate was connected to the ground of the voltage source, and the (+) terminal of the voltage source was connected to the overhead line. The overhead line was located 1.65 m above the ground plane. The length of the overhead line was 2 m. The ground plane and overhead lines were electrically disconnected. When a voltage was applied to the overhead line, an electric field was generated between the overhead line and ground plane. To examine the effects of the electric field on the proposed sensor, the proposed sensor (DUT) and a commercial electric field analyzer were placed between the overhead line and ground plane. An electric and magnetic field analyzer (EHP-50F) was used to measure the 60 Hz electric field in the vicinity of the overhead line. Data were acquired at various positions, and the details are presented in Figure 6c. Because the electric field is a vector quantity, it has a direction. The x, y, and z directions of the field analyzer are indicated in Figure 6b.

**Figure 7:**
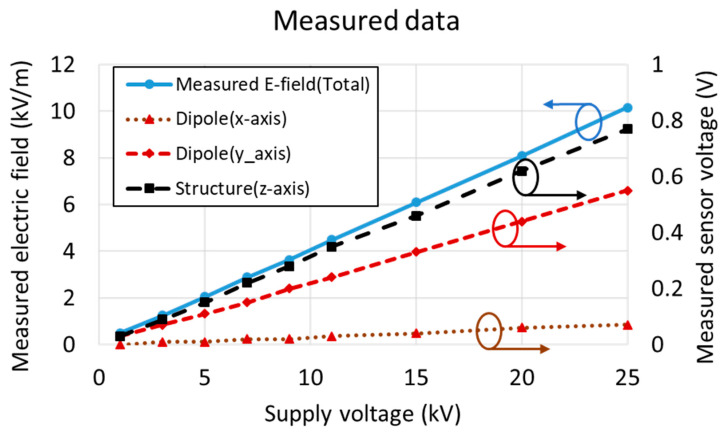
Measured values of the electric field and each axis sensor voltage in the laboratory setting with an increasing voltage applied to the overhead line.

To examine the relationship between the proposed sensor and the measured electric field, the measured electric field and sensor voltages were compared with respect to changes in the supply voltage at position *e*, as indicated in Figure 6c. Figure 7 shows the values of the measured electric field and the voltage measured by each proposed sensor when the supply voltage of the overhead line was increased from 1 to 25 kV. Over the electric field range of 0.5 to 10.1 kV/m, voltages of 0 to 0.77 V were measured by the proposed sensor. From these measured values, it can be seen that as the voltage is consistently increased, the intensity of the electric field also increases. Accordingly, the values of the voltage measured by the sensor also demonstrated a constant increase.

Next, the electric field and sensor voltage were measured from positions *a* to *f*, as indicated in Figure 6c. The results are presented in Table 4.

The proposed sensor has a structure that receives the electric field of each axis. However, since the distance between the ADC and the sensor exists when installing the sensor on the drone, lead wires are needed to electrically connect it. This lead wire also works as a part of the sensor. No matter how well the designed sensor receives only one-direction electric field, if it is manufactured, it can receive an electric field in the other direction. If the magnitude of the electric field measured in each direction is *Mx, My,* and *Mz*, and the voltage induced from the sensor in each direction is set as *Vx, Vy, Vz,* the relationship between electric field and the induced voltage can be expressed as follows.

(6) $$M_{x}=a_{11}V_{x}+a_{12}V_{y}+a_{13}V_{z}\;M_{y}=a_{21}V_{x}+a_{22}V_{y}+a_{23}V_{z}\;M_{z}=a_{31}V_{x}+a_{32}V_{y}+a_{33}V_{z}$$

(7) $$\bar{M}=A\bar{V}$$

where the matrix ***A*** is weighting matrix. If the coefficient of ***A*** is determined, the electric field in each direction can be calculated from the voltage of the sensor. Using this, the magnitude of the total electric field can be calculated from the following equation.

(8) $$M_{Total}=\sqrt{M_{x}^{2}+M_{y}^{2}+M_{z}^{2}}$$

where, we named $M_{Total}$ as the compensated sensor value. 

To obtain the A matrix, the measured sensor values and electric field values from three points *d*, *e*, and *f* in Figure 6c were used. If there are measured values for three different points, ***V*** vector and ***M*** vector can be expressed in the form of a 3 × 3 matrix, the A matrix can be calculated from the following formula.

(9) $$A=MV^{-1}$$

The compensated sensor values extracted from the calculated ***A*** are shown on the right side of Table 4. It can be seen that the measured electric field and sensor value at positions *a* to *f* show similar trends. For positions *d* to *f*, which are located at a distance of 0.7 m in the horizontal direction from positions *a* to *c*, the values of the electric field are observed to not be significantly affected by the height. The measured electric field and sensor voltage decrease as it approaches point *f*. 

**Figure 8:**
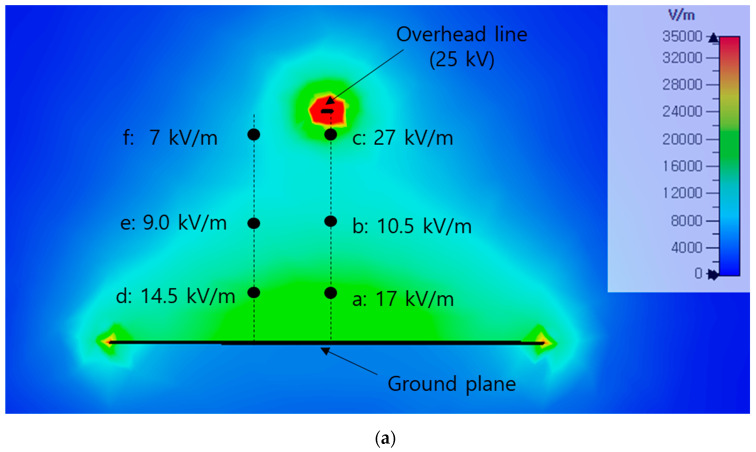

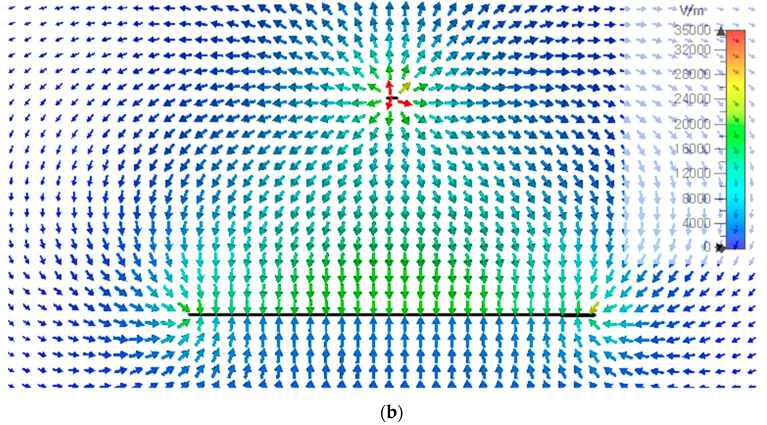
Distribution of the simulated electric field: (**a**) contour plot; (**b**) vector plot.

Figure 8 shows the results of a simulation of the electric field intensity when a voltage of 25 kV is applied to the laboratory setup. It can be seen that the simulated electric field intensity shows a similar trend to the measured electric field intensity for each position of the sensor. The simulation result has a slightly lower electric field value than the measurement result. Considering the positions at which some discrepancies from the measured values are observed, these differences may have occurred because the simulation was unable to the accurately model the metal apparatus at these locations (i.e., the pantograph, supply power cable, etc.).

**Figure 9:**
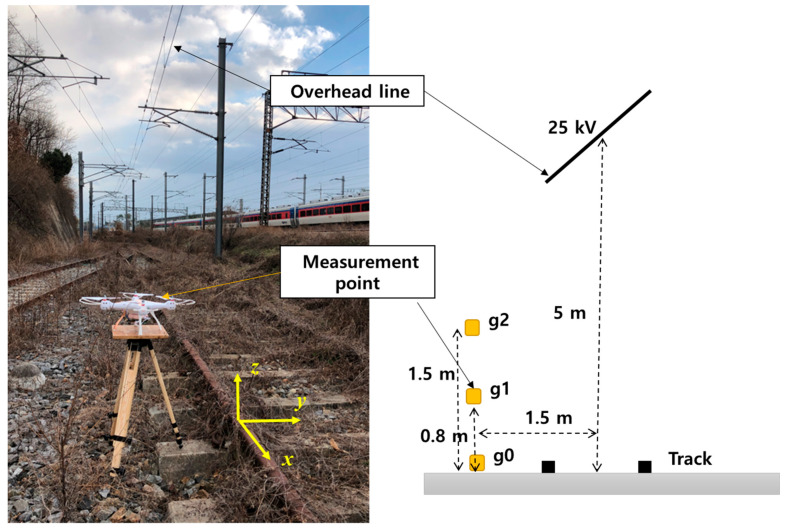
Location of the measurement point of the sensor in a railway environment with overhead lines.

**Figure 10:**
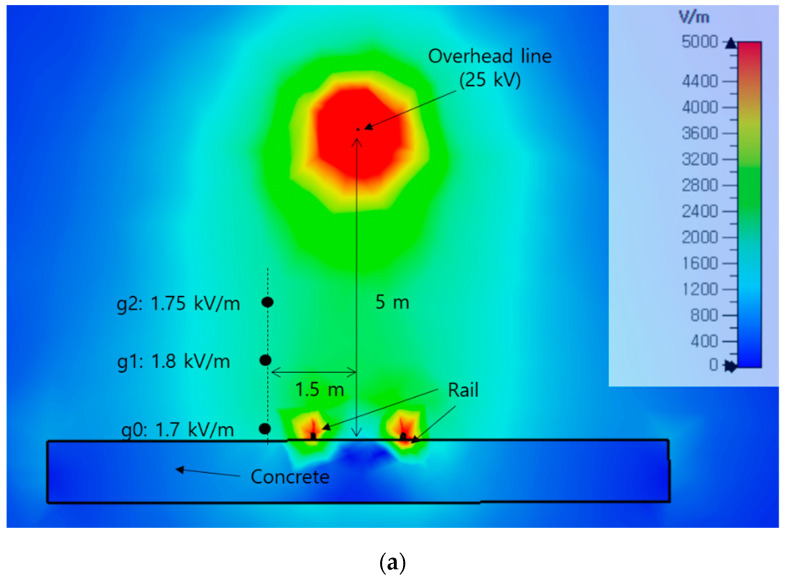

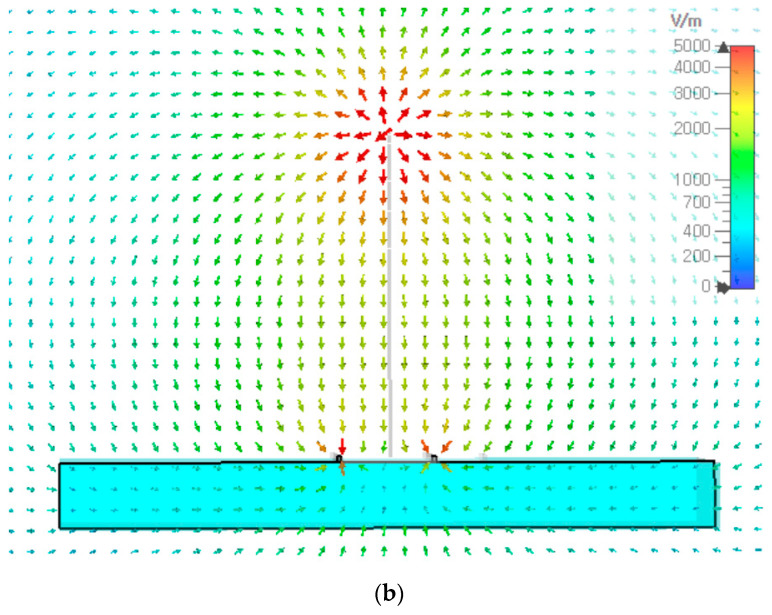
Simulated electric field distribution in the railway environment: (**a**) contour plot; (**b**) vector plot.

Figure 9 shows a real-world railway environment used to obtain experimental measurements. The electric field and the voltage of the sensor were measured at three points located 1.5 m from the center of the railway line. The measurement results are shown in Table 5. The compensated sensor values are slightly higher than the measured electric field. However, the trend does not deviate. In the railway environment, the voltage applied to the overhead line was 25 kV, and the distribution of the simulated electric field in this experimental environment is shown in Figure 10. The value of the simulated electric field at point g1 is 1.8 kV/m, which is lower than the measured value of 4.05 kV/m. In the real-world environment, several overhead lines with an applied voltage in the vicinity of the measurement positions may affect the obtained measurements, thereby resulting in higher values of the measured electric field.

From the above results, it can be seen that the proposed sensor produces compensated sensor values that are 7 V or greater in a large electric field environment. Therefore, if a system is designed such that it provides an indication when the measured sensor value is above a pre-determined value, a drone may be able to detect approaching the high-voltage lines. In this study, the measured voltage of the sensor was made in units of 0.01 V. Because this unit is not affected by ambient noise. If this value is roughly converted to an electric field value, it corresponds to 0.3 kV/m. Therefore, it can be seen that the proposed sensor has the resolution to distinguish an electric field of 0.3 kV/m.

## 4. Conclusions

In this study, a sensor for use in drones to measure electric fields in their surroundings is proposed to prevent contact between drones and high-voltage power lines. Because the voltage measurements achieved by the sensor are determined by the length of the sensor, a method for incorporating the body of the drone as a part of the sensor was proposed. In the structure of the proposed sensor, two dipole antenna structures were used to detect the electric field in the horizontal direction. In addition, to measure the electric field in the vertical direction, a structure using a metal plate and a metal post was proposed.

The voltage induced in the proposed sensor with respect to the electric field was measured in both laboratory and real-world railway environments. In the laboratory environment, the voltage of the proposed sensor was measured to be 0 to 0.77 V for an electric field in the range of 0.5 to 10.1 kV/m. In the real-world railway environment, the measured electric field was 4.05 kV/m at a height of 0.8 m, and the compensated sensor value was 4.54. The sensor proposed in this study may be used such that drones can detect high-voltage power lines through measurements of high electric fields.

## Figures and Tables

**Table 1:** Induced voltage for the different orientations of the sensors.

Sensors		Measured Voltage (V)
Small dipole antenna	Vertical	127
	Horizontal	0.4
Parallel palate capacitor	Vertical	1
	Horizontal	108

**Table 2:** Induced voltage for the proposed sensors.

Direction of E-Field	Proposed Sensors	Simulated Sensor Voltage (V)
x-axis	Dipole for x-axis	177
	Dipole for y-axis	0.5
	Structure for z-axis	0.5
y-axis	Dipole for x-axis	0.7
	Dipole for y-axis	180
	Structure for z-axis	114
z-axis	Dipole for x-axis	4.5
	Dipole for y-axis	8.5
	Structure for z-axis	111

**Table 3:** Induced voltage for different size of structure for z-axis.

Metal Length of Drone’s Leg (cm)	Width and Length of Upper Metal Plate (cm)	Simulated z-axis Sensor Voltage (V)	
		for y-axis E-Field	for z-axis E-Field
11	2 × 2	84	81
11	5 × 5	114	111
11	8 × 8	123	137
8	5 × 5	92	92
5	5 × 5	75	74

**Table 4:** Comparison of the measured electric field and sensor voltage with respect to the position of the measurement points.

Position	Measured Electric Field (kV/m)				Measured Sensor Voltage (V)			Compensated Sensor Value
	x-Direction	y-Direction	z-Direction	Total	Dipole (x)	Dipole (y)	Structure (z)	
*a*	1.40	2.99	20.12	20.39	0.53	0.68	1.69	20.35209
*b*	3.70	1.68	14.09	14.66	0.26	0.50	1.20	14.11101
*c*	1.81	3.03	30.31	30.52	0.96	0.75	2.27	28.78331
*d*	0.68	0.60	15.99	16.01	0.41	0.69	1.33	16.01497
*e*	2.46	3.84	9.08	10.16	0.07	0.55	0.77	10.16209
*f*	2.27	6.31	2.49	7.16	0.16	0.40	0.15	7.155126

**Table 5:** Measured values of the electric field and sensor voltage in the railway environment.

Position	Measured Electric Field (kV/m)				Measured Sensor Voltage (V)			Compensated Sensor Value
	x-Direction	y-Direction	z-Direction	Total	Dipole (x)	Dipole (y)	Structure (z)	
g0	0.69	0.35	2.83	2.94	0.13	0.14	0.30	3.684274
g1	0.71	0.68	3.93	4.05	0.20	0.17	0.36	4.540885
g2	0.69	0.79	4.05	4.18	0.17	0.16	0.39	4.829291

## Data Availability

Not applicable.
